# Functional and kinetics of two efficient phenylalanine ammonia lyase from *Pyrus bretschneideri*

**DOI:** 10.1186/s12870-023-04586-0

**Published:** 2023-12-02

**Authors:** Guohui Li, Cheng Song, Muhammad Aamir Manzoor, Daoyuan Li, Yunpeng Cao, Yongping Cai

**Affiliations:** 1 Anhui Engineering Research Center for Eco-Agriculture of Traditional Chinese Medicine, Anhui Provincial Key Laboratory for Quality Evaluation and Improvement of Traditional Chinese Medicine, College of Biological and Pharmaceutical Engineering, West Anhui University, Lu’an, 237012 China; 2 Department of Plant Science, School of Agriculture and Biology, Shanghai Jiao Tong University, Shanghai, China; 3 CAS Key Laboratory of Plant Germplasm Enhancement and Specialty Agriculture, Wuhan Botanical Garden, Chinese Academy of Sciences, Wuhan, 430074 China; 4 Anhui Agricultural University, Hefei, 230036 China

**Keywords:** Phenylalanine ammonia lyase (PAL), Lignin, Kinetics, *Pyrus bretschneideri*

## Abstract

**Background:**

The enzyme phenylalanine ammonia lyase (PAL) controls the transition from primary to secondary metabolism by converting L-phenylalanine (L-Phe) to cinnamic acid. However, the function of PAL in pear plants (*Pyrus bretschneideri*) has not yet been fully elucidated.

**Results:**

We identified three *PAL* genes (*PbPAL1*, *PbPAL2* and *PbPAL3*) from the pear genome by exploring pear genome databases. The evolutionary tree revealed that three *PbPAL*s were classified into one group. We expressed PbPAL1 and PbPAL2 recombinant proteins, and the purified PbPAL1 and PbPAL2 proteins showed strict substrate specificity for L-Phe, no activity toward L-Tyr in vitro, and modest changes in kinetics and enzyme characteristics. Furthermore, overexpression of *PbAL1* and *PbPAL1*-RNAi, respectively, and resulted in significant changes in stone cell and lignin contents in pear fruits. The results of yeast one-hybrid (Y1H) assays that *PbWLIM1* could bind to the conserved PAL box in the *PbPAL* promoter and regulate the transcription level of *PbPAL2.*

**Conclusions:**

Our findings not only showed PbPAL’s potential role in lignin biosynthesis but also laid the foundation for future studies on the regulation of lignin synthesis and stone cell development in pear fruit utilizing molecular biology approaches.

**Supplementary Information:**

The online version contains supplementary material available at 10.1186/s12870-023-04586-0.

## Background

PAL is the first key enzyme in the biosynthesis phenylpropanoid pathway, and has been extensively investigated. It is the rate-limiting step in phenylpropanoid metabolism, catalyzing the conversion of L-phenylalanine to cinnamic acid to connect the primary and secondary metabolism [1]. PAL does not have a single Km value, and its active center has an electrophilic center consisting of a dehydroalanyl group [2]. In general, PAL coding genes contain one to five members [3, 4]. The PAL gene family contains more than five members in several plants, including *Eucalyptus grandis* [5] and watermelon (*Citrus lanatus*) [6].

The expression and activity of the PAL enzyme determine the flux through the phenylpropane pathway and the production rate of phenylpropane compounds [7]. Previous studies have shown that PAL is the key gene for lignin production [8]. Consequently, a better understanding of plant PAL expression and activity will aid in controlling the synthesis of phenylpropanes via molecular mechanisms, hence regulating the synthesis of lignin. For example, overexpression of *AtPAL1* and *AtPAL2* led to an increase in lignin content in *Arabidopsis*. In addition, the lignin content of *Arabidopsis* with *pal1* and *pal2* double mutants was dramatically reduced [9]. *RcPAL* is a crucial gene in *Ricinus communis* lignin production that can be induced under mechanical damage stress. When *RcPAL* is overexpressed, it significantly increases lignin content while reducing plant height. However, this the gene is silenced, it significantly reduces lignin synthesis [10].

*PAL* expression is regulated by the transcription factors MYB, LIM and NTS [11, 12]. In *Arabidopsis*, PAL activity is targeted for degradation to through the ubiquitination of Kelch F-box (KFB) protein after transcription, decreasing lignin biosynthesis [13]. The LIM transcription factor family has been widely studied in animals, and it has been gradually found to have many important functions in plants in recent years [14]. Research on the overexpression of found that four LIMs have been confirmed to regulate lignin synthesis or secondary wall lignification, they are respectively tobacco *NtLIM1*, upland cotton *GhWLIM1a*, poplar *PtaGLIM1a* and *Eucalyptus camaldulensis EcLIM1* [15–17]. Recently, GhXLIM6 isolated from upland cotton was shown to affect cotton fiber development by regulating cellulose synthesis genes, which may be due to the functional differentiation of LIM domain sequence changes [18]. Cheng et al. [19] identified 14 nonredundant PbLIMs in the pear genome, of which 2LIMs are only distributed in the WLIM1, WLIM2 and PLIM2 subclasses, and PbWLIM1a and PbWLIM1b belong to the WLIM1 subclass. The expression levels of PbWLIM in different developmental stages of pear fruit showed that two members of PbWLIM1, PbWLIM1a and PbWLIM1b, were probably closely related to the lignin synthesis of pear fruit [19].

Pear is grown worldwide as a commercial fruit crop pear. Cai et al. [20] reported that the ‘Dangshan Su’ pear (*Pyrus bretschneideri* cv. Dangshan Su) is a commercial fruit crop species in China (Dangshan County, Anhui Province, China). The size and content of the stone cell mass are major factors for determining the quality of pear fruit [21]. Lignin is one of the primary components of pear stone cells [22], and the activities of key enzymes in the lignin biosynthesis pathway are regulated via the expression of corresponding genes [23].

Phenylpropane, the first substrate in lignin synthesis in pear fruit, is produced by the shikimate pathway, and cinnamic acid is formed by phenylpropane catalyzed by the PAL enzyme [24, 25]. However, the PALs in pear plants remain unclear despite their crucial roles in the formation of procyanidins, flavonols, and their phenolic derivatives. In this study, three genes encoding PALs were identified in the pear genome. Previous research results showed that *PbPAL1* and *PbPAL2* are key genes involved in lignification in pear fruits [8]. Consequently, *PbPAL1* and *PbPAL2* were intensively characterized. Enzymatic activities were examined via heterologous expression in vitro, and the effects of overexpression and RNAi of *PbPAL1* on lignin content were examined in pear fruits. We hoped to clarify its role in lignin synthesis in pear stone cells by in vivo and in vitro experiments.

## Results

### Phylogenetic tree analysis among members of the PbPAL family

**Fig. 1:**
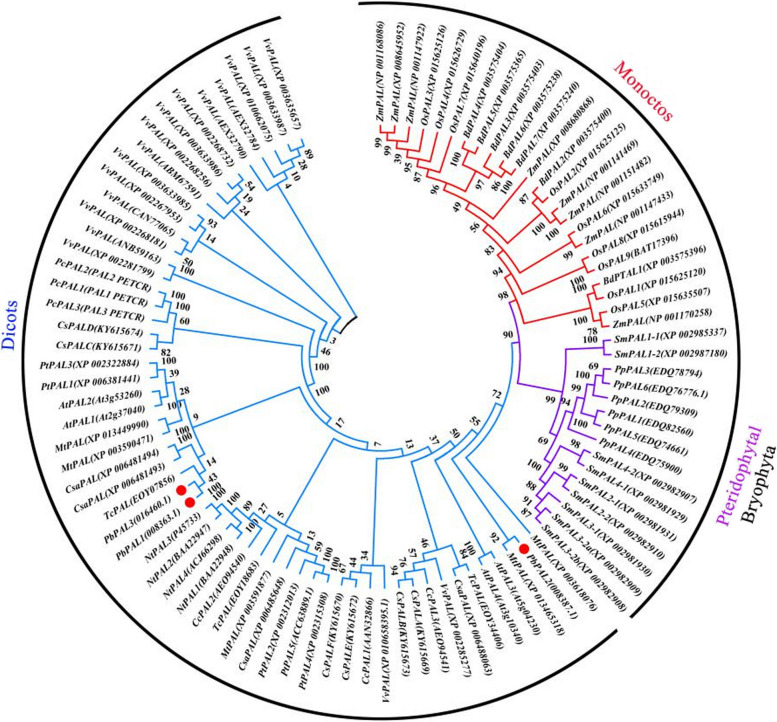
Phylogenetic analysis of *PAL*s. Note: A total of 96 PAL genes were divided into three clades from *Selaginella lamariscina*, *Vitis vinifera*, *Cucumis sativus*, *Zea mas*, *Nicotiana tabacum*, *Arabidopsis thaliana* etc.

A recent study showed that *NnPAL1*, an ancient member of the PAL gene family, may be the origin of PAL diversity in angiosperm evolution [26]. To explore the evolutionary relationships of *PbPAL*, a phylogenetic tree was created based on the protein sequences of other species. These PALs are divided into three categories, including ferns/bryophytes, monocotyledons and dicotyledons (Fig. 1). The evolutionary tree can be divided into dicotyledons, monocotyledons, gymnosperms and angiosperms, which are similar to those previously reported [27]. The cladistic structure of the phylogenetic tree is usually matched with traditional taxonomic classification. In this study, the PALs of monocotyledons and dicotyledons are found in each subfamily of the PAL family of angiosperms, whereas other evolutionary branches are divided into angiosperms and gymnosperms. This analysis clearly showed that *PbPAL* is phylogenetically into the angiosperm-type PAL family and the dicotyledon subfamily.

### Functional prediction of PbPAL proteins in each phylogenetic group

**Fig. 2:**
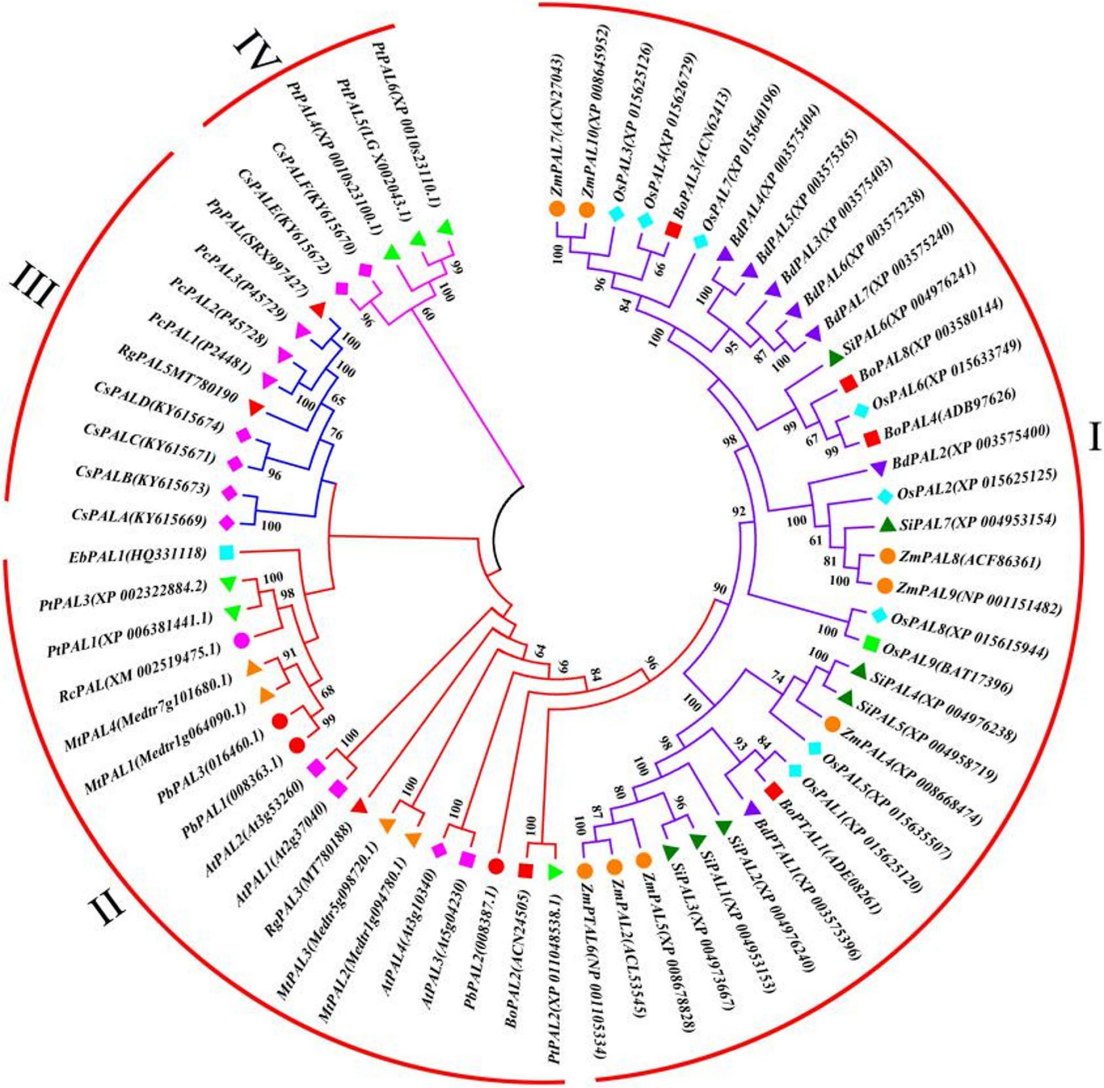
Phylogenetic analysis of *PbPAL* and characterized *PAL*s from other plant species. Note: A total of 67 PAL genes were divided into four clades from *Brachypodium dispachyon*,*Vitis vinifera*, *Cucumis sativus*, *Zea mas*, *Nicotiana tabacum*, *Arabidopsis thaliana*, *Populus euphratica*, *Ricinus communis* etc.

In our study, a phylogenetic tree was built based on the PALs whose functions (in lignin biology, flavonoid synthesis, and coumarin synthesis) have been confirmed, including the members *PbPAL* gene family in pear and 64 other species, to further analyze the potential roles of the *PbPAL* gene family in the phenylpropane pathway (Fig. 2). According to the results of the phylogenetic tree, PALs are divided into four phylogenetic groups (Groups I-IV). Three *PbPAL*s exist in the group II, including *AtPAL1*, *EbPAL1*, *RcPAL* and other genes closely related to lignin synthesis. It is speculated that *PbPAL1*, 2 and *3* may have similar biological functions and participate in the synthesis of lignin in pear fruit.

### Expression and enzyme properties of PbPAL proteins in vitro

Recombinant histidine-labeled PbPAL1 and PAL2 proteins were successfully expressed in the pET-22b vector. We determined that the fusion protein was expressed after inoculation and induction of the successfully transformed strain by 12% SDS-PAGE (Additional file 1: Fig. S1A). The molecular weights of the recombinant proteins PbPAL1 and PbPAL2 were approximately 79.0 kDa (including the label), which was close to the molecular weight of the target protein (Additional file 1: Fig. S1B).

**Fig. 3:**
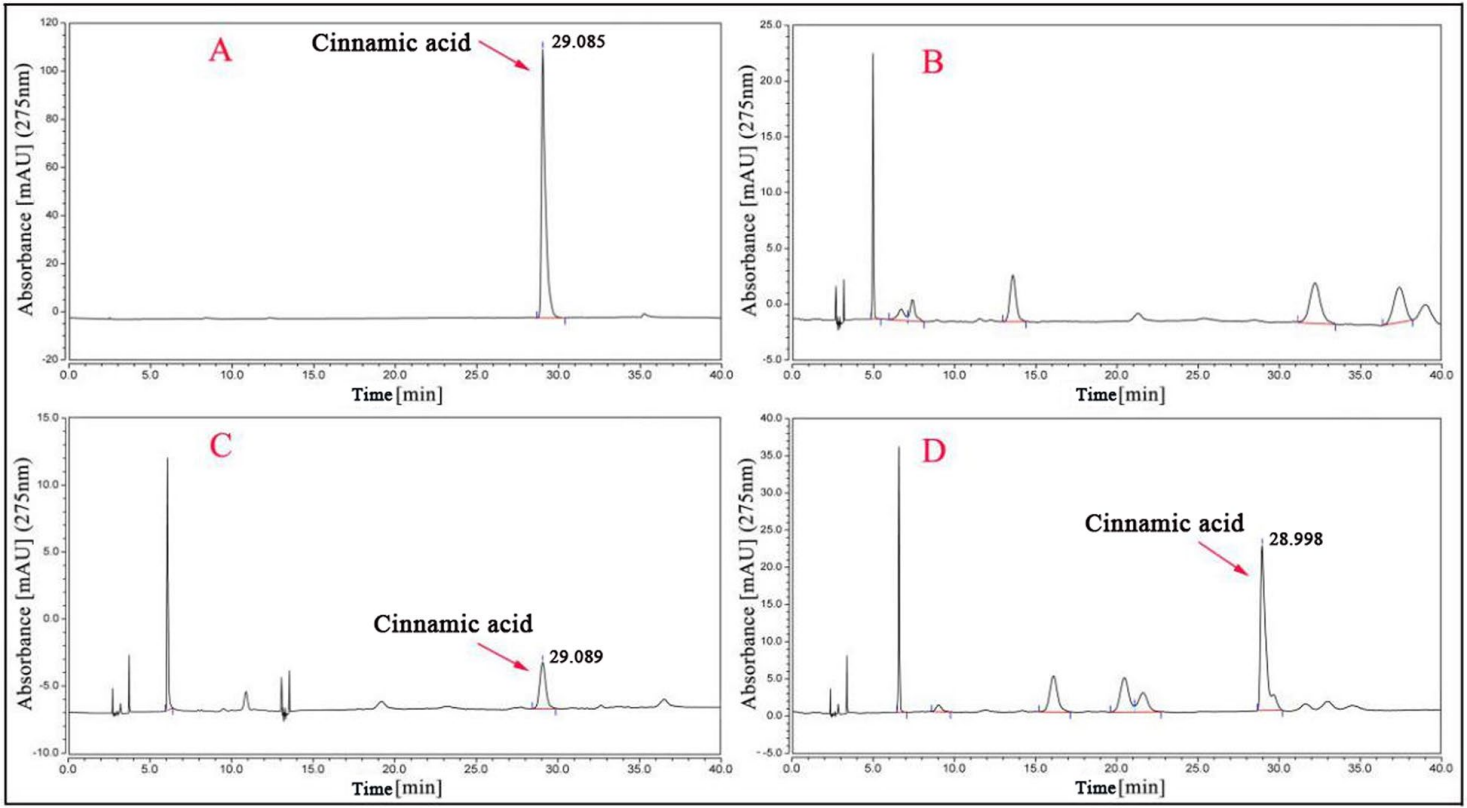
HPLC chromatograms for the initial assay of PbPAL enzymatic activity. **A** Cinnamic acid standard. **B** The crude extracts of the naïve strain without supplementation with L-Phe. **C** The crude extracts of the expression strain without L-Phe. **D** The crude extracts of the expression strain with 0.6 mM L-Phe

Native *E. coli* produces a basic level of major metabolites to maintain its survival. Therefore, in the expression strain, even if no foreign substrate is added to the crude extract, the recombinant PAL protein can also use the internal L-Phe to produce some cinnamic acid [28]. Therefore, this study first measured PAL activity in the absence of L-Phe (Fig. 3). Recombinant PAL protein can create cinnamic acid without the addition of exogenous L-Phe, although the amount produced is negligible compared to the amount produced with the addition of exogenous L-Phe.

**Fig. 4:**
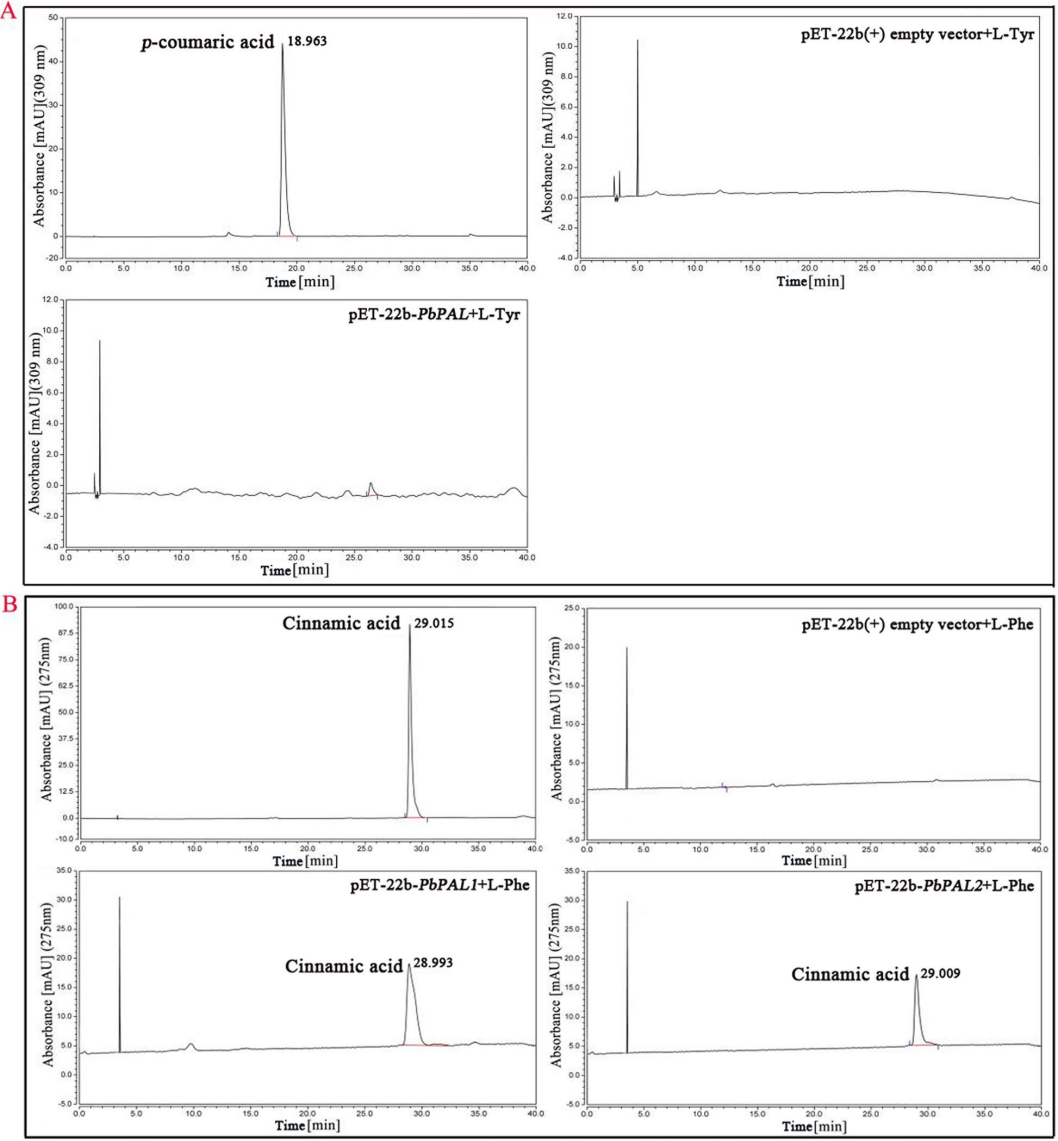
HPLC analysis of enzyme reaction products from the incubation of PbPAL1 and PbPAL2 infusion protein. Note: **A** Analysis of protein products of PbPAL1 and PbPAL2 using L-Tyr as substrate, **B** Analysis of protein products of PbPAL1 and PbPAL2 using L-Phe as substrate

The deamination of aromatic amino acids were catalyzed by aromatic amino acid lyase (AAAL). In this experiment, the amino acid alignment showed that PbPAL1 and PbPAL2 are AAAL, possibly HAL or PAL (Additional file 1: Fig. S2). Therefore, PbPAL1 and PbPAL2 were first reacted with L-Tyr, and the deamination of L-Tyr directly resulted in the generation of *p*-coumaric acid. Unfortunately, the HPLC failed to reveal the appropriate product (Fig. 4A), proving that neither PbPAL1 nor PbPAL2 possess the HAL function. The background interference is eliminated when L-Phe is used as the substrate, as shown in Fig. 4B, since the broken cell carrying the empty plasmid’s crude enzyme solution cannot catalyze L-Phe. In addition, when L-Phe was used as the substrate, the HPLC results showed that both PbPAL1 and PbPAL2 recombinant protein molecules could transform L-Phe into less polar products in approximately 29.00 min. The retention time was almost identical to the cinnamic acid standard value.

### Enzyme kinetic analysis of PbPAL1 and PbPAL2 proteins

To understand the biochemical characterizations of PAL1 and PAL2, the enzyme assays were carried out with L-Phe as the substrate. The optimal pH (8.5 ~ 9.0) and temperature (45 ℃ ~ 55 ℃) of the two purified PAL proteins were roughly the same (Additional file 1: Fig. S3), which was in good agreement with the results in *Arabidopsis* [29] and *Camellia sinensis* [28]. Moreover, the apparent kinetic parameters of two purified proteins, PbPAL1 and PbPAL2, were determined using L-Phe as substrate. Compared the PbPAL2 protein, the PbPAL1 protein showed a higher Kcat/Km (higher catalytic efficiency) for L-Phe (Table 1). Based on the above results, this study found that the two PbPAL proteins showed strict substrate specificity for L-Phe but had no catalytic activity for L-Tyr toward PbPAL1 had higher catalytic efficiency.

**Table 1:** Biochemical characterization of PAL1 and PAL2, including the pH optima, temperature optima, and kinetic parameters

Enzyme	*Km* (M)	*Vmax* (nKat/mg^−1^)	*Kcat* (S^−1^)	*Kcat/Km* (S^−1^/M^−1^)
PbPAL1	89.4	2.3	0.15	596.33
PbPAL2	116.3	3.6	0.28	415.36

### Functional verification of *PbPAL1* in pear fruit

To further explore the role of *PbPAL* in the development of pear fruit stone cells transiently overexpressing *PbPAL1* (*PbPAL1*-OE) and RNAi (*PbPAL1*-RNAi), and each treatmentwas used to transform 40 pear fruits. Pear fruits were collected immediately after, and detect the expression level of *PbPAL1* in the fruits was detected (Additional file 1: Fig. S4). In pear *PbPAL1*-OE fruit, the transcript level of *PbPAL1* was 1.592 times higher than that of the control fruit. However, the expression level of *PbPAL1* in *PbPAL1*-RNAi fruit was only 53.82% of that in the control fruit. Therefore, transient overexpression and RNAi of *PbPAL1* in pear fruits can effectively increase or inhibit the expression of *PbPAL1*.

**Fig. 5:**
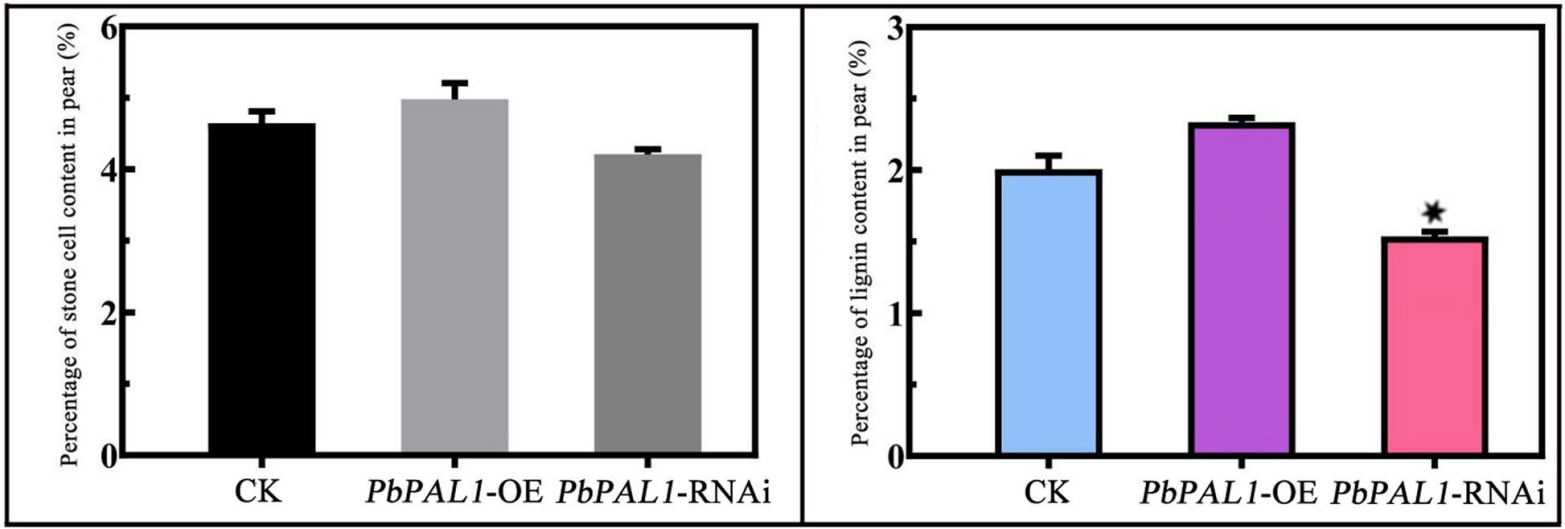
Determination of stone cells and lignin content in *PbPAL1* overexpression and RNAi pear fruit. Note: Error bars represent the mean ± SD (*n* = 3), *indicates a significant difference (*P* < 0.05)

As shown in Fig. 5, the content of stone cells in pear *PbPAL1*-OE fruit (4.98%) was higher than that of the control group; In *PbPAL1*-RNAi fruit, the content of stone cells was only 4.21%, which was lower than that of the control fruit (4.67%). At the same time, the lignin content of pear fruit overexpressing *PbPAL1* was 12.1% higher than that of the control fruit. The lignin content of *PbPAL1*-RNAi pear fruit was only 75.36% of that of the control fruit (the lignin content of the control fruit was 1.99%). In general, the contents of stone cells and lignin in pear fruits overexpressing *PbPAL1* were higher than those in the control group, but they did not reach statistical significance. However, the content of lignin and stone cells was reduced in the *PbPAL1*-RNAi pear fruit, and the level of lignin reached a substantial difference.

**Fig. 6:**
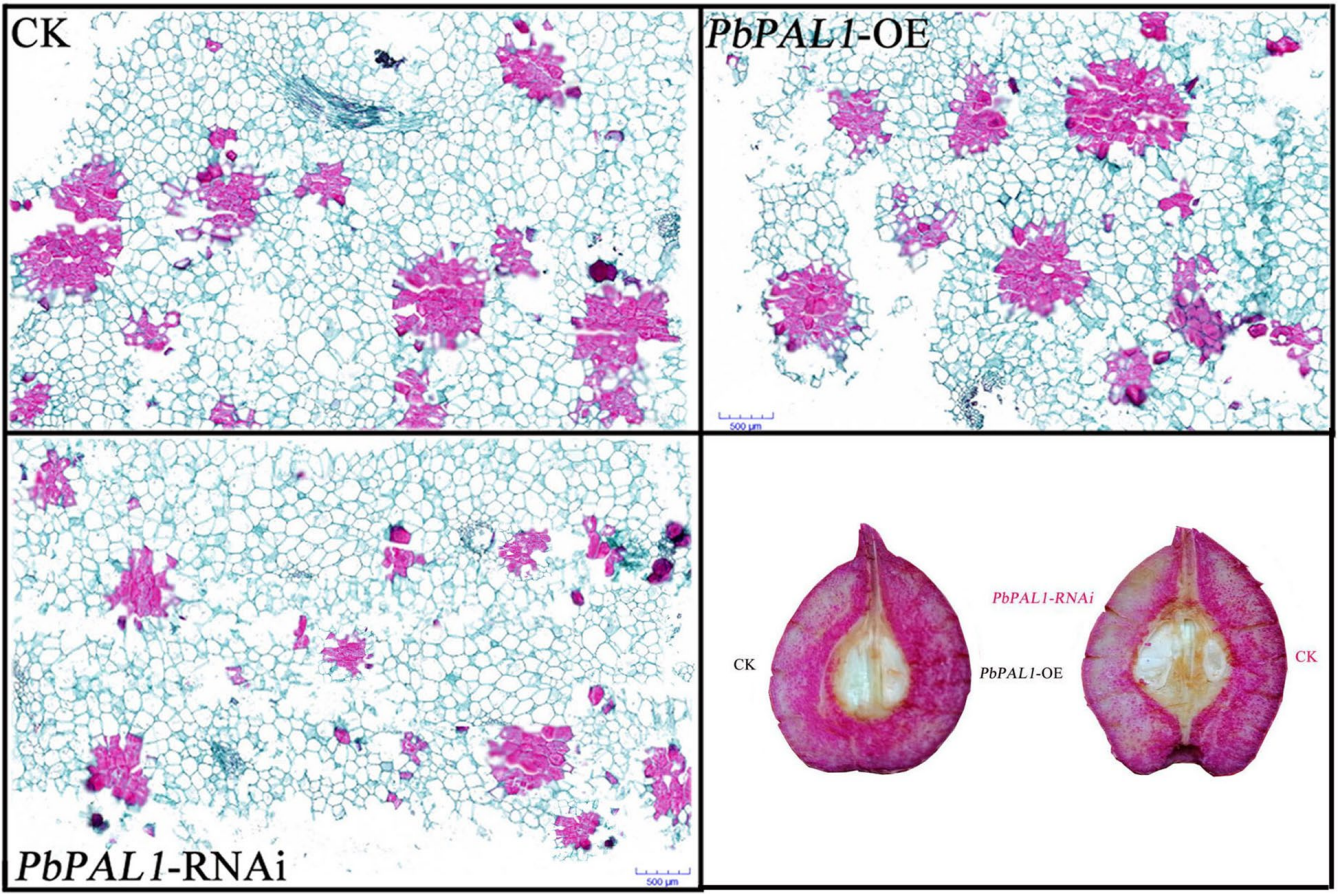
Observation of saffron fixation green and phloroglucinol staining of transient transformation pear fruit

To further understand the role of *PbPAL* in responses to the development of stone cells in pear fruit, we performed the histochemical staining to observe the stone cell differences in the transverse section of the pear fruit. The safranine fixation green staining results of *PbPAL1*-OE and *PbPAL1*-RNAi pear fruits and phloroglucinol staining were similar (Fig. 6). Compared with the fruits of the control group, more stone cells in *PbPAL1*-OE pear fruits were stained red and were not only accumulated near the fruit core but were also distributed in the pulp area, and the number and size of stone cell clusters in the fruit increased. The staining intensity of stone cells in *PbPAL1*-RNAi pear fruit was low in PbPAL1-RNAi pear fruit, the location of stone cells around the fruit core and pulp area was greatly reduced compared to the control, and the quantity and size of stone cell clusters were also significantly reduced.

### Metabolite accumulation analyses in transgenic pear fruit

**Fig. 7:**
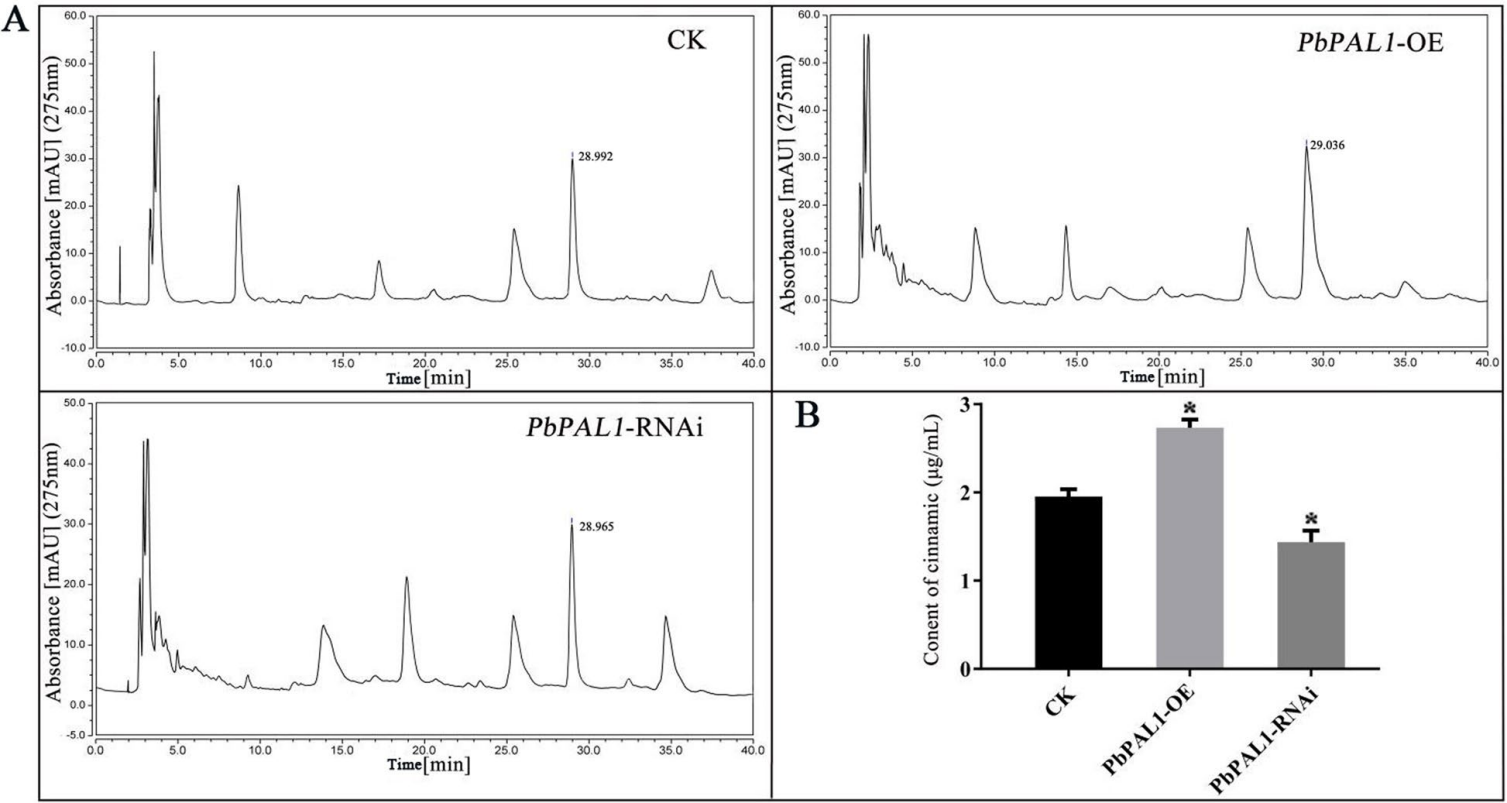
Determination of cinnamic acid content in *PbPAL1* overexpression and RNAi of pear fruit. Note: **A** HPLC chromatograms for cinnamic acid content; **B** determination of cinnamic acid content, *indicates a significant difference (*P* < 0.05)

Cinnamic acid is an important intermediate in lignin synthesis in pear fruit and is closely related to lignin synthesis and the formation of stone cells [30]. The retention time of cinnamic acid in pear control fruit was 28.992 min. The retention time of cinnamic acid in *PbPAL1*-OE and *PbPAL1*-RNAi fruits was 29.036 min and 28.965 min, respectively (Fig. 7A). The content of cinnamic acid in *PbPAL1*-OE pear fruit increased significantly, reaching 2.791 μg/mL. Compared with the control pear fruit, the content of cinnamic acid in *PbPAL1*-RNAi fruit decreased significantly by 0.817 μg/mL (Fig. 7B). This showed that PbPAL1 can promote the production of cinnamic acid in the synthesis of lignin in pear fruit to improve the accumulation level of lignin.

### Expression profile of key genes in lignin synthesis in pear fruit

**Fig. 8:**
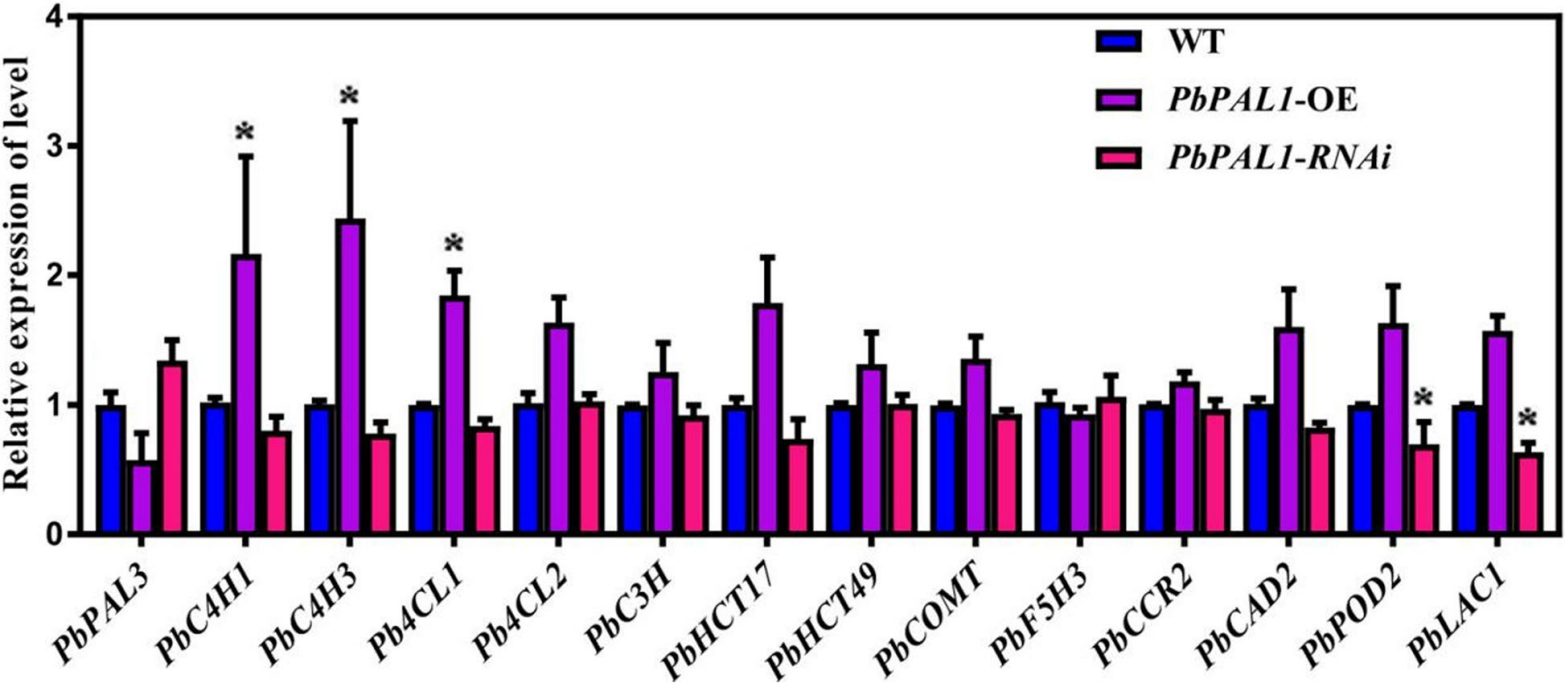
Transient overexpression of *PbPAL1* and RNAi expression pattern analysis of key genes for lignin synthesis in pear fruit. Note: *indicates a significant difference (*P* < 0.05)

Overexpression of *PbPAL1* (*PbPAL1*-OE) in pear fruit can activate the expression of most of the key enzyme coding genes for lignin synthesis (such as *PbC4H1*, *3*, *Pb4CL1*, *PbHCT17*), making its expression level 1.7 ~ 2.3 times higher than that of the control (Fig. 8). However, in *PbPAL1*-RNAi pear fruit, the expression of most genes, including *PbPOD2* and *PbLAC1*, was significantly inhibited (the expression amount was only 48.1%-91.3% of the control group), while the expression level of *PbPAL3* was increased. The change in the *PbPAL1* expression level had little effect on the *PbC3H*, *PbCCR2* and *PbHCT49* expression levels, and there was no substantial change in the expression levels of these three genes. In addition, *PbPOD2* and *PbLAC1*, two genes related to lignin polymerization, were significantly affected by changes in the expression level of *PbPAL1*. The expression of *PbPOD2* and *PbLAC1* was inhibited when the expression of *PbPAL1* decreased and the lignin content decreased.

### *PbWLIM* specifically binds PAL-box elements to regulate *PbPAL* gene expression

**Fig. 9:**
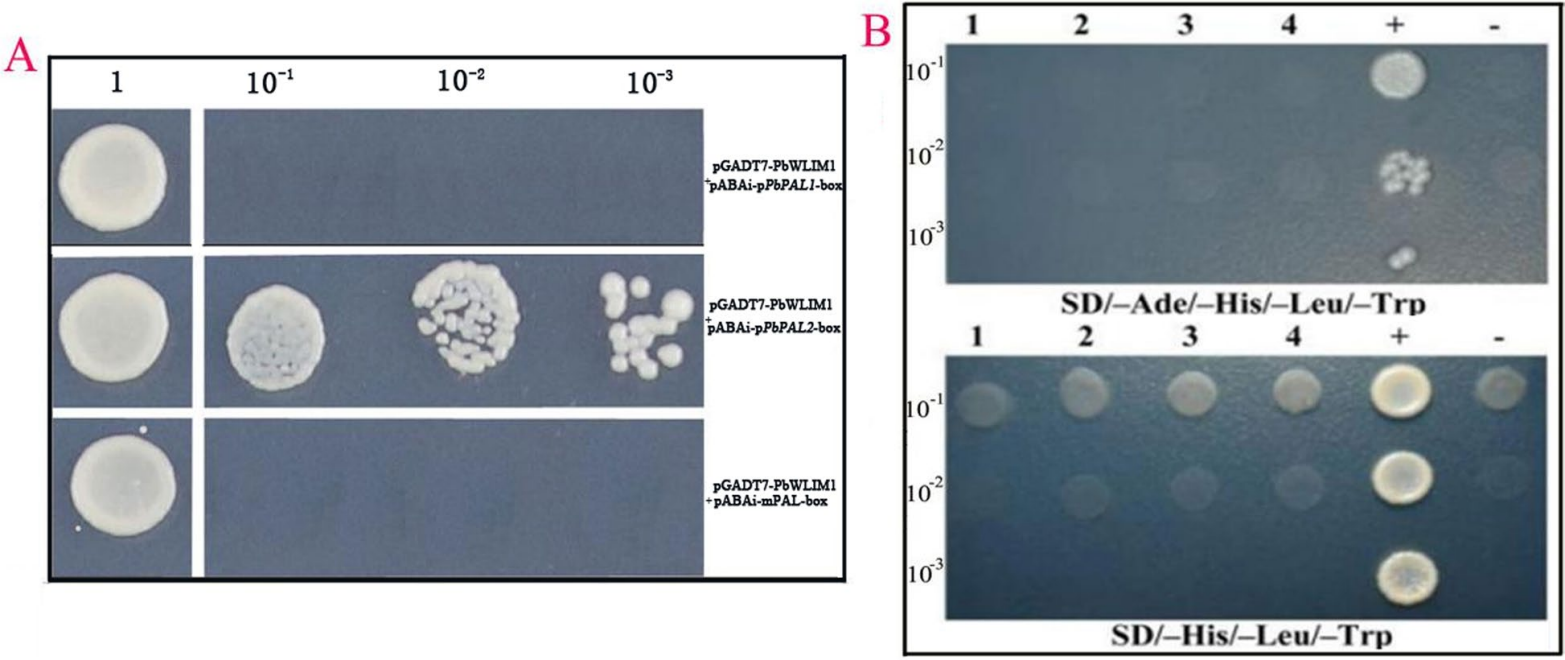
Yeast one-hybrid assay to verify the binding activity of PbWLIM1 and PAL-box element. Note: **A** PbWLIM1 can bind to the conserved PAL box in the PbPAL promoter, 1: Stock solution, 10^–1^_,_ 10^–2^ and 10^–3^: the bacterial solution is diluted 10, 100 and 1000 times; **B** Detection of the activation activity of pGBKT7-PbWLIM1a/b in yeast, 1,2,3,4 represents the adjustment of 4 bacterial plaques, + and—represent positive and negative controls respectively, 1, 10^–2^ and 10^–3^: the bacterial solution is diluted 10, 100 and 1000 times

At present, research on *NtLIM1* and *GhWLIM1a* is relatively in depth, revealing that LIM transcription factors can selectively bind PAL-box elements in the promoter of lignin metabolism-related genes [CCAC (A/C) AN (A/C) N (C/T) (A/C)], thereby activating their transcription [16]. To explore whether *PbWLIM* can regulate the transcription level of *PbPA*L by binding with the PAL box, our study screened the potential binding sites of *PbWLIM* in the *PbPAL* family gene promoter (Additional file 2: Table S1). Our results showed that there were conserved PAL-box elements in the promoters of some members of the *PbPAL* family. To further prove that *PbPAL* is a target gene regulated by *PbWLIM1*, yeast single hybridization (Y1H) was performed, and the results (Fig. 9) showed that *PbWLIM1* could combine with the conserved PAL box in the *PbPAL2* promoter (Fig. 9A). However, pGBKT7-*PbWLIM1a* and pGBKT7-*PbWLIM1b* did not show transactivation activity in yeast (Fig. 9B). It is speculated that *PbWLIM1a/1b* may play a role in the nucleus by combining with other transcription regulators to form a complex, thus realizing the function of regulating gene expression.

## Discussion

Phenylpropane compounds, including lignin, anthocyanins, plant antitoxins, and condensed tannins, play important roles in plant development. All of these secondary metabolites are produced by plants through the phenylpropane route. Phenylalanine ammonia lyase (PAL) plays an important role in the general phenylpropanoid pathway. It catalyzes the conversion of L-phenylalanine to cinnamic acid, which is the rate-limiting step of the phenylpropane pathway. Therefore, PAL controls the metabolic flux in the phenylpropanoid pathway [7]. In recent years, although PAL family members have been identified in some angiosperms, research on the PAL family in fruit trees, especially in pears, has not been reported. Here, three *PAL*s were identified in the pear genome, and to clarify their functions in lignin synthesis in pear fruit, bioinformatics and functional analyses were carried out systematically.

In several plants, PAL proteins are encoded via a multigene family, which has different numbers of members in different plants. For example, there are 3 members in *Nelumbo nucifera*, 4 members in *Arabidopsis*, 8 *PAL*s in *Brachypodium dispachyon*, and 9 *PAL*s in the eucalyptus genome [26, 31, 32]. Because PAL exists in a polygene family, it has been proposed that different PAL isozymes may be responsible for producing different metabolites. During the evolutionary process of PAL, lineage-specific duplication took place in *Arabidopsis*, *Populus euphratica* and *Selaginella lamariscina*, which is a common phenomenon promoting the diversity of polygenic families. PALs were classified into three categories after the monocotyledon and dicotyledon split, with no gene expansion events, such as grapes (*Vitis vinifera*) and cucumbers (*Cucumis sativus*) [33]. *PbPAL1*, *PbPAL2*, and *PbPAL3* were all found in the same subgroup (Fig. 1), indicating that *PbPAL*s were created via gene duplication.

The 67 PAL members of pear can be divided into four phylogenetic groups (Fig. 2). In Group I, there are a large number of PALs of other species, including multiple PTALs with dual functions, such as BdPTAL1, ZmPTAL and BoPTAL3, which seem to have dual functions and can effectively utilize L-Phe and L-Tyr [31]. In group III, *PpPAL* participates in the biosynthesis of coumarin in Peucedanum [34], indicating that this subfamily may contain PAL members involved in coumarin biosynthesis. Group IV contains *CsPALE* and *CsPALF,* which may be involved in the synthesis of flavonoids and lignin [28], indicating that PAL members of this family may be involved in multiple pathways of phenylpropanoid metabolism.

In plants, PALs are aggregated ferns/bryophytes and monocotyledons, such as *ZmPAL*, *NnPAL1* and *BdPAL1*, which seem to have dual functions and can effectively utilize L-Phe and L-Tyr [26, 31]. In tobacco, L-Phe has been proven to be a real physiological substrate, and the three isoforms of PAL proteins showed significant dynamic differences [6]. In this study, we used a prokaryotic expression system to verify the function of PbPAL1 and PbPAL2 (Fig. 4). The results showed that the two recombinant proteins expressed in vitro both possessed enzymatic activity, and they showed slight differences in enzyme kinetics. The catalytic efficiency of PbPAL1 was relatively high. These critical proteins were functional, and PbPAL1 may play a more vital role in the biology of pear solid lignin, laying the groundwork for increasing pear fruit quality at the molecular level in the future.

Some enzymes have a very clear function in the manufacture of lignin. It has occasionally been possible to alter the genetic makeup of plant lignin or decrease lignin content by genetic manipulation [35]. Previous studies found that although *pal1* and *pal2* single mutants did not have obvious phenotypes during growth and development, the phenotypes of *pal1* and *pal2* double mutants changed, including sterility, significant reduction of lignin accumulation, and changes in the ultrastructure of secondary cell walls [36]. In addition, recent studies have found that *RcPAL1* is a key gene in ricin lignin biosynthesis. When it is upregulated, it will significantly increase lignin content and plant height [10]. Unfortunately, little is known about PAL in lignin synthesis in pear fruit. To verify the actual function of pear PALs, *PbPAL1* was instantaneously overexpressed and subjected to RNAi in pear fruit to further analyze its role in pear fruit lignin synthesis. Our results showed that the instantaneous overexpression of *PbPAL1* and RNAi led to changes in stone cells and lignin content of pear fruit (Figs. 5 and 6). However, *PAL* is the most upstream gene of the phenylpropanoid pathway, and it has little inhibitory effect lignin synthesis. In the future, it is hoped that RNAi of multiple phenylpropanoid pathway genes (*PAL*, *C4H*, *4CL*, etc.) can effectively inhibit the synthesis of lignin in pear fruit and improve the quality of pear fruit.

In recent years, the integration of metabolomics studies has been used as an important means to identify crucial genes involved in the synthesis of target compounds [37, 38]. The identification of phenolic compounds involved in lignin biosynthesis has been performed mainly through liquid chromatography [35]. Cinnamic acid is essential to the production of lignin, similar to any other intermediate [20]. In this paper, based on cinnamic acid as the product of the PAL target enzyme, we analyzed the metabolites resulting from *PbPAL1* overexpression and RNAi in pear fruit via HPLC. Our results showed that increased expression of *PbPAL* could lead to increased conversion of phenylalanine to cinnamic acid (Fig. 7), which may be used for the downstream lignin synthesis pathway. This finding implies that researchers can change the content of cinnamic acid to regulate the synthesis of lignin and the development of stone cells in pear fruit by regulating the expression level of *PbPAL* in pear fruit, ultimately improving the quality of pear fruit.

Although poplar *PtaGLIM1a* and *Eucalyptus camaldulensis EcLIM1* have been indicated to affect plant lignin synthesis, their specific regulatory mechanisms have not been reported [15, 17]. In this study, *PbWLIM1* can combine with the conserved PAL-box element in the *PbPAL* promoter to regulate the expression of the *PbPAL* gene (Fig. 9A). Interestingly, these two genes have no transactivation activity in yeast (Fig. 9B). Coincidentally, millet *SiWLIM2b* cannot activate the reporter gene in yeast, but overexpression of this gene can significantly enhance the transcription level of phenylpropanoid metabolism-related genes in plants [39]. Recent studies have also found that the transcription factor of sand pear PpHY5 (ELONGATEDHYPOCOTYL 5) has no transactivation activity in yeast, but dual-LUC can activate the expression of the target gene PpMYB10. When PpHY5 is cotransformed with its interacting protein PpBBX18 (B-box protein), the activity of the PpMYB10 promoter can be greatly improved [40]. It can be inferred that since the dual-LUC experiment is carried out in plants, LIM can ‘recruit’ certain interacting proteins to form a complex in plants to regulate gene expression or have a more efficient regulatory effect. Because there is no homologous gene to that encoding this interacting protein in yeast, *PbWLIM1* does not show transactivation activity. PbWLIM1a/1b acts as a ‘bridge’ connecting promoters and other proteins in the regulatory complex, or both DNA binding function and trans activation activity. This series of problems needs to be further studied.

## Conclusions

In summary, a total of three full-length *PAL* genes were identified in pear, designated PbPAL1-PAL3, and two *PAL* genes have been cloned. The resulting proteins both showed strict substrate specificity toward L-Phe and subtle differences in kinetics and enzymatic properties. The analysis of enzyme kinetics showed that *PbPAL1* may be the key gene for lignin synthesis in pear fruit. Furthermore, the instantaneous overexpression and RNAi of *PbPAL1* in pear fruit provided evidence for the involvement of this gene in lignin metabolism. The results of Y1H showed that *PbWLIM1* may regulate the transcription level of *PbPAL.* Our research provides a new strategy for reducing the lignin content and stone cell size in pear fruit.

## Materials and methods

### Plant materials

Fruit samples (39 days after flowering (DAF)) were collected from 40-year-old *Pyrus bretschneideri* cv. Dangshan Su, which was grown in an orchard in Dangshan, Anhui, China. All samples were stored at -80 °C for subsequent use.

### Identification of PAL family members

In this study, the genome data of Chinese white pear were obtained from the pear genome (http://gigadb.org/dataset/100083) [41]. The obtained amino acid sequence of the whole genome of pear used the ‘BioEdit’software to establish a local database and used the *AtPAL1* (At2G37040) and *AtPAL2* (At3G53260) sequences as query sequences. Then, all candidate *PbPAL*s were verified using Pfam and SMART3 to confirm that they contained the core domains [42, 43]. Finally, all potentially redundant PAL sequences were discarded according to the sequence alignments.

### Construction of PAL phylogenetic tree

Sequence analysis of *PbPAL* gene family members of pear by was performed with the ClusterW tool in MEGA 11 with bootstrap analysis (1000 replicates) and compared with PAL sequences of other species collected through the NCBI database. After the sequence alignment was completed, the neighbor-joining (N-J) method was used to construct the phylogenetic tree to obtain the evolutionary analysis of the PAL gene family of pear.

### Construction of the plant expression vector

The *PbPAL1* and *PbPAL2* genes were PCR amplified from pear fruit. The primers are listed in Table S2 and contain restriction sites (*Bgl* II and *Spe* I). Subsequently, the amplicon was inserted into the vector pCAMBIA1304 (GenBank: AF234300.1) by T4 DNA ligase. Finally, the resulting vectors were integrated into Agrobacterium tumefaciens strain EHA105.

### RNA extraction and qRT-PCR analysis

Total RNA was extracted from the collected samples using TRIzol reagent (Invitrogen). Then, we used a one-step RT-PCR kit (Takara, China) to reverse transcribe RNA into first-strand cDNA according to the manufacturer's instructions. The *tubulin g*ene was used as an internal control [41], and the gene-specific primers (Additional file 2: Table S3) of each PbPAL gene were designed using Beacon Designer 7.9 software. qRT‒PCR was performed with a CFX96 Touch™ Real-Time PCR Detection System (BIO-RAD). The relative expression level was calculated by the 2^−△△ CT^ method [44].

### Instantaneous expression of PbPAL1 in pear fruit

Fresh pear fruits at 39 DAF were selected for transient expression. A syringe was used to take up the Agrobacterium culture liquid and slowly and evenly inject it into the pear fruit, fully inject 50% (50 μL) of culture liquid. A total of 35 ~ 45 pear fruits were injected with each kind of Agrobacterium infection solution. After 7 DAF of living culture on fruit trees, the fruits were removed and stored.

### Determination of stone cell and lignin content in instantaneously transformed pear fruit

The stone cell content of pear fruit was determined according to the method described by Cheng [45]: the flesh (5.0 g) was frozen at -20 °C for 24 h and then centrifuged at 20 000 rpm/min for 3 min. The homogeneous pulp was incubated in water, and then the upper suspension was discarded. This process was repeated 4–5 times, and the collected stone cells were dried and weighed in the oven. The content of stone cells was calculated as follows: (weight of stone cells/weight of pulp × 100% = stone cell content (%)).

According to the method reported by Yan [46], the lignin content of pear fruit was determined with some adjustments. The instantaneously transformed pear fruit grinding powder (0.02 g) was collected, filtered using a 20 mesh sieve and placed into a 10 mL frosted glass tube. Subsequently, 2 mL of 25% bromoacetyl and glacial acetic acid was added, and the tube was sealed with a glass stopper. The mixture was allowed to react in a 70 °C water bath for approximately 30 min, and then 2 mL of 2 M NaOH was added to terminate the reaction. The mixture was transferred to a volumetric flask and diluted to 100 mL with glacial acetic acid. Finally, the absorbance (ABS) of the solution was measured at 280 nm.

### Observation of pear fruit tissue section staining

The pear fruit was peeled immediately after transformation. A fruit tissue sample from near the fruit core was washed and placed it FAA fixative solution. The sample was placed under vacuum and allowed to stand for 12 h. After fixation, the samples were embedded in wax blocks, placed on an automatic microtome for sectioning, and then stained with safranine and green for observation. Then, 1.0 mol/L hydrochloric acid in 1.0% phloroglucinol solution was added. The results were observed and photographed.

### Determination of cinnamic acid content in pear fruit

Samples were obtained by quartering. An electronic balance was used to weigh 100 mg of pear flesh, which was then frozen with liquid nitrogen. Next, 80% ethanol solution was added at a material:liquid ratio of 1:15. It was ground with a ball mill, ultrasonicated at 45 ℃ for 50 min, and centrifuged at 12 000 r/min for 10 min. The supernatant was removed, and 1 mL of 100% chromatographic grade methanol was added for redissolution. The solution was passed through 0.22 μM organic phase membrane filtration, with three biological replicates for each group of samples. Metabolites were analyzed according to the Proestos and Komaitis methods [47].

### Heterologous expression and purification of recombinant PbPALs

The full-length PbPAL1 and PbPAL2 cDNAs were cloned into plasmid pET-22b encoding water-soluble maltose-binding protein (MBP) upstream of the inserted site, followed by the heat shock method, and transfected into *Escherichia coli* BL21(DE3) cells. The purity of the His-tag-fused PbPALs was assessed by analyzing the total protein on 12% SDS‒PAGE gels. The purified proteins were used for further enzymatic assays.

### Enzymatic assays of PbPALs

The activity of PbPAL1 and 2 was analyzed according to the method of Wu et al. [28] with minor modifications. The purified recombinant PAL proteins (6 μg) were incubated with 100 μL of reaction buffer consisting of 0.6 mM L-Phe (100%, Aladdin) or L-Tyr (100%, Aladdin) and 100 mM Tris HCl buffer (pH 8.0) (or boiling for 5 min as a control). After incubation at 50 °C for 30 min, the reaction was terminated with an equal volume of 100% methanol.

To determine the optimal reaction pH, assays were performed at 50 °C, and 2 μg each of NaAC-HAC (100 mM, pH 5.0 ~ 6.0), Tris HCl (100 mM, pH 7.0 ~ 8.0), and Na_2_CO_3_-NaHCO_3_ (100 mM, pH 9.0 ~ 11.0) was added. The purified recombinant PbPAL protein was reacted for 15 min. Then, to determine the most appropriate temperature, the mixture was incubated at pH 8.0 for 15 min with the temperature varying from 20 °C to 80 °C. To measure the Km and Vmax of the recombinant protein, the concentration of L-Phe in Tris HCl (pH 7.5) was changed from 2 μM to 250 μM, and the mixture was incubated at 30 °C for 15 min.

Products were subjected to the HPLC elution (150 × 2.1 mm, 1.7 μm) procedure as follows: starting from 10% eluent B (100% methanol), the linear gradient was 15%—30% B for 0 min ~ 5 min, 30%-45% B for 5 min ~ 35 min, 45%-10% B for 35 min ~ 39 min, and then stopped at 40 min.

### Yeast one-hybrid assay

For the Y1H assay, by cloning the target (*PbPAL1* and *PbPAL2*) gene into the vector pAbAi, the pBait AbAi bait carrier was obtained, pBait AbAi was loaded into yeast, and the self-activation of the bait carrier was detected on the AbA plate. The total RNA of pear fruit was extracted by TIANGEN’s RNAprep Pure plant Total RNA Extraction Kit, the poly A + mRNA was enriched by the NucleoTrap mRNA Kit, and the first strand was synthesized by oligo dT (CDS III). Long-distance PCR (LD-PCR) was used to amplify and synthesize double-stranded (ds) cDNA, and ds cDNA was purified by a CHROMA SPIN + TE-400 column. The ORF of *PbWLIM1* was fused in-frame with the GAL4 activation domain encoded by the sequence in the pGADT7 vector to generate pGADT7-PbWLIM1. The purified ds cDNA and pGADT7 vector were cotransferred into yeast with the pBait AbAi bait vector and screened with SD medium supplemented with AbA.

### Statistical analysis

Samples were analyzed in biological triplicate, and the data are presented as mean standard deviation. Statistical signifificance was determined using one-way ANOVA and Student’s t-test (^*^P < 0.05, ^**^P < 0.01). Bars with different letters represent signifificantly different means (*P* < 0.05).

### Supplementary Information

**Additional file 1:** **Fig. S1.** A: SDS-PAGE of protein induction (M: Mark, 1: a uninduced sample, 2-3 are samples induced by PbPAL1 proteins, 4: a uninduced sample, 5-6: are samples induced by PbPAL1 PbPAL2 proteins); B: is the purification of PbPAL1 and PbPAL2 proteins (M: Mark, 1: a uninduced sample, 2-3 are samples induced by PbPAL1 proteins, 4: a uninduced sample, 5-6: are samples induced by PbPAL1 PbPAL2 proteins). **Fig. S2.** Sequence alignment of PAL amino acids in different species. **Fig. S3.** Biochemical characterization of PALa-PALf, including the pH optima and temperature optima. **Fig. S4.** Analysis of PbPAL1 expression level in overexpression and RNAi pear fruit.

**Additional file 2:** **Table S1.** Potential PbWLIM1 binding sites in the PbPAL promoter. **Table S2.** Primer sequences contained artificial restriction enzyme sites for *Bgl* II and *Spe* I. **Table S3.** Primer sequences used for qRT-PCR.

## Data Availability

The *PbPAL1* and *PbPAL2* genes sequence used in this study were available inGenBank with accession numbers of MF346686 and MF346687.

## Abbreviations

ABS: Absorbance
PCR: Polymerase chain reaction
qRT-PCR: Real Time PCR
RT-PCR: Reverse transcription PCR
RNAi: RNA interference

## References

[1] Pavel F, Liubov S, Anastasiia R, Pungin A, Tokupova E, Maslennikov P, Chupakhina G (2020). Phenylalanine and tyrosine as exogenous precursors of wheat (Triticum aestivum L) secondary metabolism through PAL-associated pathways. Plants.

[2] Mădălina EM, Diana AA, Emma ZAN, Szarvas N, Tosa MI, Paizs C, Bencze LC (2020). Fluorescent enzyme-coupled activity assay for phenylalanine ammonia-lyases. Sci Rep.

[3] Stefania T, Antonio F, Cherubino L, Romano D (2018). PAL activities in asparagus spears during storage after ammonium sulfate treatments. Postharvest Biol Tec.

[4] Ban ZJ, Luo ZH, Chen CK, Gong JY, Yuan QP, Yu LK, Yu W, Li L (2017). Cloning of *PcPAL* gene from *Pyrus communis* and characterization of its expression in two cultivars with different anthocyanin accumulation levels. J Biobased Mate Bio.

[5] Victor C, Marcal S, Charles H, Cassan-Wang H, Fevereiro P, Myburg AA, Paiva JAP, Grima-Pettenati J (2015). Genome-wide analysis of the lignin toolbox of *Eucalyptus grandis*. New Phytol.

[6] Dong SJ, Shang QM (2013). Genome-wide characterization of phenylalanine ammonia-lyase gene family in watermelon (*Citrullus lanatus*). Planta.

[7] Wang ZB, Chen X, Wang W, Kong JQ (2014). Transcriptome-wide identification and characterization of Ornithogalum saundersiae phenylalanine ammonia lyase gene family. RSC Adv.

[8] Li GH, Wang H, Cheng X, Su XQ, Zhao Y, Jiang TS, Jin Q, Lin Y, Cai YP (2019). Comparative genomic analysis of the PAL genes in five Rosaceae species and functional identification of Chinese white pear. PeerJ.

[9] Huang JL, Gu M, Lai ZB, Fan BF, Shi K, Zhou YH, Yu JQ, Chen ZX (2010). Functional analysis of the Arabidopsis PAL gene family in plant growth, development, and response to environmental stress. Plant Physiol.

[10] Lu JN, Shi YZ, Li WJ, Chen S, Wang YF, He XL, Yin XG (2019). RcPAL, a key gene in lignin biosynthesis in *Ricinus communis* L. BMC Plant Biol.

[11] Zhao Q, Dixon RA (2011). Transcriptional networks for lignin biosynthesis: more complex than we thought?. Trends Plant Sci.

[12] Zhong RQ, Ye ZH (2007). Regulation of cell wall biosynthesis. Curr Opin Plant Biol.

[13] Zhang XB, Gou MY, Liu CJ (2013). Arabidopsis kelch repeat F-Box proteins regulate phenylpropanoid biosynthesis via controlling the turnover of Phenylalanine ammonia-Lyase. Plant Cell.

[14] Srivastava V, Verma PK (2017). The plant LIM proteins: unlocking the hidden attractions. Planta.

[15] Kawaoka A, Nanto K, Ishii K, Ebinuma H (2006). Reduction of lignin content by suppression of expression of the LIM domain transcription factor in *Eucalyptus camaldulensis*. Silvae Genet.

[16] Han LB, Li YB, Wang HY, Wu XM, Li CL, Luo M, Wu SJ, Kong ZS, Pei Y, Jiao GL, Xia GX (2013). The dual functions of WLIM1a in cell elongation and secondary wall formation in developing cotton fibers. Plant Cell.

[17] Yang SH, Zhu GY, Wang CL, Chen LC, Song YJ, Wang JH (2017). Regulation of secondary xylem formation in young hybrid poplars by modifying the expression levels of the PtaGLIMa gene. Mol Breed.

[18] Li Y, Wang NN, Wang Y, Liu D, Gao Y, Li L, Li XB (2018). The cotton XLIM protein (GhXLIM6) is required for fiber development via maintaining dynamic F-actin cytoskeleton and modulating cellulose biosynthesis. Plant J.

[19] Cheng X, Li GH, Abdullah M, Zhang JY, Jiang TS, Jin Q, Zhao H, Cai YP (2019). Molecular identification, phylogenomic characterization and expression patterns analysis of the LIM (LIN-11, Isl1 and MEC-3 domains) gene family in pear (*Pyrus bretschneideri*) reveal its potential role in lignin metabolism. Gene.

[20] Cai YP, Li GQ, Nie JQ, Lin Y, Nie F, Zhang JY, Xu YL (2010). Study of the structure and biosynthetic pathway of lignin in stone cells of pear. Sci Hortic Amsterdam.

[21] Jin Q, Yan CC, Qiu JX, Zhang N, Lin Y, Cai YP (2013). Structural characterization and deposition of stone cell lignin in Dangshan Su pear. Sci Hortic.

[22] Brahem M, Renard CC, Gouble B, Bureau S, Le BC (2017). Characterization of tissue specific differences in cell wall polysaccharides of ripe and overripe pear fruit. Carbohydr Polym.

[23] Huang LN, Wu GB, Zhang S, Kuang FY, Chen FH (2019). The identification and functional verification of the cinnamate 4-hydroxylase gene from wax apple fruit and its role in lignin biosynthesis during nitric oxide-delayed postharvest cottony softening. Postharvest Biol Technol.

[24] Irisarri P, Zhebentyayeva T, Errea P, Pina A (2016). Difffferential expression of phenylalanine ammonia lyase (PAL) genes implies distinct roles in development of graft incompatibility symptoms in prunus. Sci Hortic.

[25] Zhang WW, Li JB, Feng X, Tang Y, Cheng SY, Cao FL (2016). Isolation and characterization of a phenylalanine ammonia-lyase gene (PAL) promoter from *Ginkgo biloba* and its regulation of gene expression in transgenic tobacco plants. RSC Adv.

[26] Wu ZH, Gui ST, Wang SZ, Ding Y (2014). Molecular evolution and functional characterisation of an ancient phenylalanine ammonia-lyase gene (*NnPAL1*) from *Nelumbo nucifera*: novel insight into the evolution of the PAL family in angiosperms. BMC Evol Bio.

[27] Zhu QL, Xie XR, Lin HX (2015). Isolation and functional characterization of a phenylalanine ammonia-lyase Gene (SsPAL1) from coleus (Solenostemon scutellarioides L.). Molecules.

[28] Wu YL, Wang WZ, Li YZ, Dai XL, Ma GL, Xing DW, Zhu MQ, Gao LP, Xia T (2017). Six phenylalanine ammonia-lyases from Camellia sinensis: evolution, expression, and kinetics. Plant Physiol Biochem.

[29] Cochrane F, Davin LB, Lewis NG (2004). The Arabidopsis phenylalanine ammonia lyase gene family: kinetic characterization of the four PAL isoforms. Phytochem.

[30] Tao ST, Zhang H, Zhang SL (2009). Anatomy, ultrastructure and lignin distribution of stone cells in two *Pyrus* species. Plant Sci.

[31] Barros J, Serrani-Yarce JC, Chen F, Baxter D, Venables BJ, Dixon RA (2016). Role of bifunctional ammonia-lyase in grass cell wall biosynthesis. Nat plants.

[32] Cass CL, Peraldi A, Dowd PF, Mottiar Y, Santoro N, Karlen SD, Bukhman YV, Foster CE, Thrower N, Bruno LC, Moskvin OV, Johnson ET, Willhoit ME, Phutane M, Ralph J, Mansfield SD, Nicholson P, Sedbrook JC (2015). Effects of PHENYLALANINE AMMONIA LYASE (PAL) knockdown on cell wall composition, biomass digestibility, and biotic and abiotic stress responses in Brachypodium. J Exp Bot.

[33] Shang QM, Li L, Dong CJ (2012). Multiple tandem duplication of the phenylalanine ammonia-lyase genes in *Cucumis sativus* L. Plant.

[34] Sui ZW, Luo J, Yao RL, Huang CL, Zhao YC, Kong LY (2019). Functional characterization and correlation analysis of phenylalanine ammonia-lyase (PAL) in coumarin biosynthesis from *Peucedanum praeruptorum* Dunn. Phytochem.

[35] Weng JK, Chapple C (2010). The origin and evolution of lignin biosynthesis. New Phytol.

[36] Rohde A, Morreel K, Ralph J, Goeminne G, Hostyn V, De Rycke R, Kushnir S, Van Doorsselaere J, Joseleau JP, Vuylsteke M, Van Driessche G, Van Beeumen J, Messens E, Boerjan W (2004). Molecular phenotyping of the *pal1* and *pal2* mutants of *Arabidopsis thaliana* reveals far-reaching consequences on phenylpropanoid, amino acid, and carbohydrate metabolism. Plant Cell.

[37] Supaporn B, Bancha M, Sang-Kyu L, Jong-Seong J, James R, Ketudat C (2018). Demonstration of monolignol β-glucosidase activity of rice Os4BGlu14, Os4BGlu16 and Os4BGlu18 in *Arabidopsis thaliana* bglu45 mutant. Plant Physiol Biochem.

[38] Dima O, Morreel K, Vanholme B, Kim H, Ralph J, Boerjan W (2015). Small glycosylated lignin oligomers are stored in *Arabidopsis* leaf vacuoles. Plant Cell.

[39] Yang R, Chen M, Sun JC, Yu Y, Min DH, Chen J, Xu ZS, Zhou YB, Ma YZ, Zhang XH (2019). Genome-wide analysis of LIM family genes in foxtail millet (Setaria italica L.) and characterization of the role of SiWLIM2b in drought tolerance. Int J Mol Sci.

[40] Bai SL, Tao RY, Yin L, Ni JB, Yang QS, Yan XH, Yang FP, Guo XP, Li HX, Teng YW (2019). Two B-box proteins, PpBBX18 and PpBBX21, antagonistically regulate anthocyanin biosynthesis via competitive association with *Pyrus pyrifolia* ELONGATED HYPOCOTYL 5 in the peel of pear fruit. Plant J.

[41] Wu J, Wang ZW, Shi ZB, Zhang S, Ming R, Zhu SL, Khan MA, Zhang SL (2013). The genome of the pear (Pyrus bretschneideri Rehd.). Genome Res..

[42] Letunic I, Bork P (2018). 20 years of the SMART protein domain annotation resource. Nucleic Acids Res.

[43] Mistry J, Chuguransky S, Williams L, Qureshi M, Salazar GA, Sonnhammer ELL, Tosatto SCE, Paladin L, Raj S, Richardson LJ (2021). Pfam: The protein families database in 2021. Nucleic Acids Res.

[44] Garrido JMG, Morcillo RJL, Rodrıguez JAM, Bote JAO (2010). Variations in the mycorrhization characteristics in roots of wild-type and ABA-defificient tomato are accompanied by specifific transcriptomic alterations. Mol Plant Micr Int.

[45] Cheng X, Li ML, Li DH, Zhang JY, Jin Q, Sheng LL, Cai YP, Lin Y (2017). Characterization and analysis of *CCR* and *CAD* gene families at the whole-genome level for lignin synthesis of stone cells in pear (*Pyrus bretschneideri*) fruit. Biol Open.

[46] Yan CC, Yin M, Zhang N, Jin Q, Fang Z, Lin Y, Cai YP (2014). Stone cell distribution and lignin structure in various pear varieties. Sci Hortic.

[47] Proestos C, Komaitis M (2013). Analysis of naturally occurring phenolic compounds in aromatic plants by RP-HPLC coupled to diode array detector (DAD) and GC-MS after silylation. Foods.

