# Supplementary material for: Functional and kinetics of two efficient phenylalanine ammonia lyase from Pyrus bretschneideri
Source: BMC Plant Biol. 2023 Dec 2;23:612. doi: 10.1186/s12870-023-04586-0 (PMC10693048; doi:10.1186/s12870-023-04586-0)
Supplement: Supplementary file 1 — Additional file 1: Fig. S1. A: SDS-PAGE of protein induction (M: Mark, 1: a uninduced sample, 2-3 are samples induced by PbPAL1 proteins, 4: a uninduced sample, 5-6: are samples induced by PbPAL1 PbPAL2 proteins); B: is the purification of PbPAL1 and PbPAL2 proteins (M: Mark, 1: a uninduced sample, 2-3 are samples induced by PbPAL1 proteins, 4: a uninduced sample, 5-6: are samples induced by PbPAL1 PbPAL2 proteins). Fig. S2. Sequence alignment of PAL amino acids in different species. Fig. S3. Biochemical characterization of PALa-PALf, including the pH optima and temperature optima. Fig. S4. Analysis of PbPAL1 expression level in overexpression and RNAi pear fruit. [file 12870_2023_4586_MOESM1_ESM.pdf]

Additional file:1

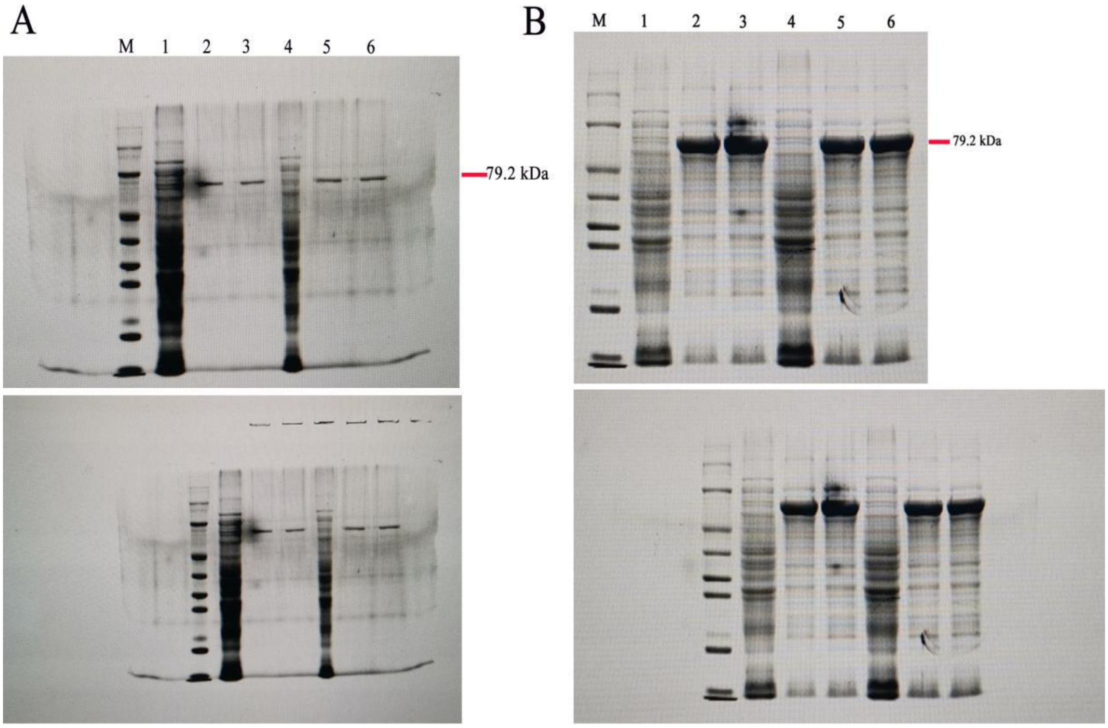

Figure. S1 Soluble analysis of recombinant pET-22b-PbPAL1/2

A: SDS-PAGE of protein induction (M: Mark, 1: a uninduced sample, 2-3 are samples induced by PbPAL1 proteins, 4: a uninduced sample, 5-6: are samples induced by PbPAL1 PbPAL2 proteins); B: is the purification of PbPAL1 and PbPAL2 proteins (M: Mark, 1: a uninduced sample, 2-3 are samples induced by PbPAL1 proteins, 4: a uninduced sample, 5-6: are samples induced by PbPAL1 PbPAL2 proteins)

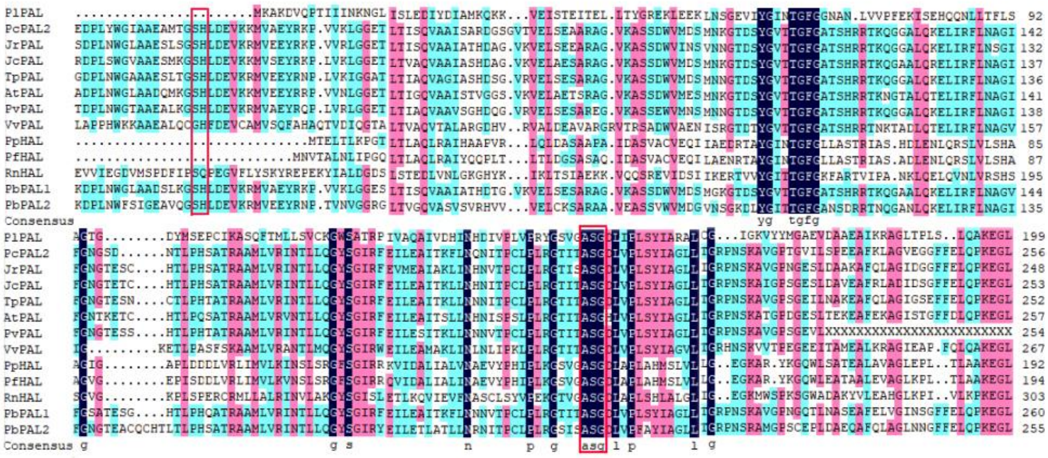

Figure. S2 Sequence alignment of PAL amino acids in different species

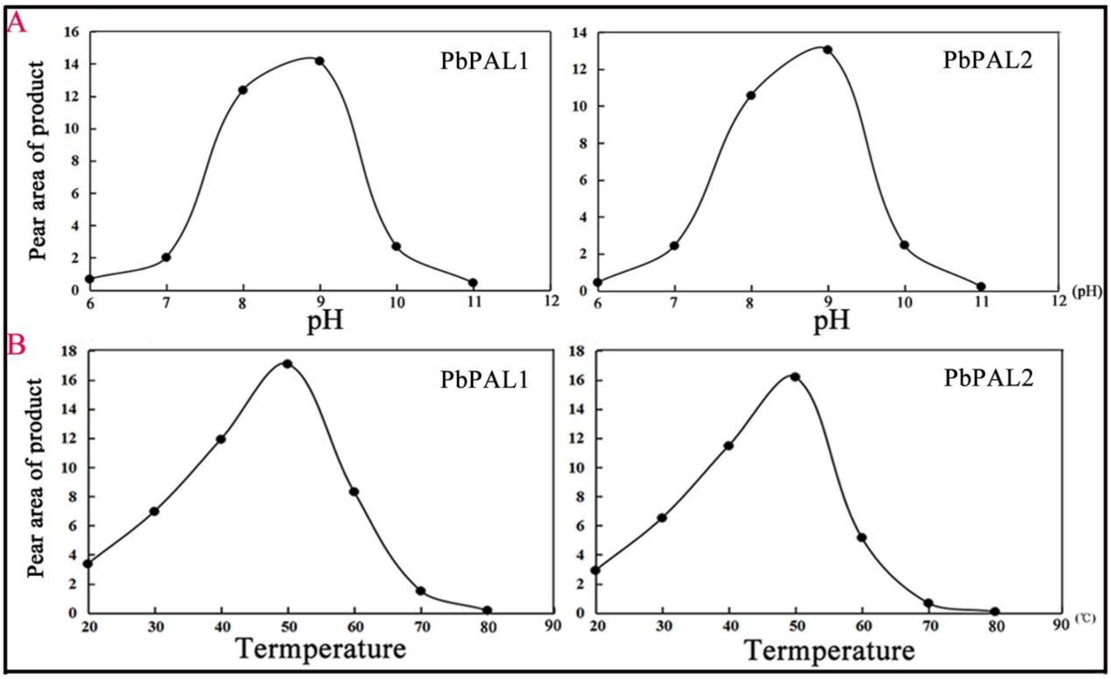

Figure. S3 The optimum pH and temperature of the two purified PAL proteins

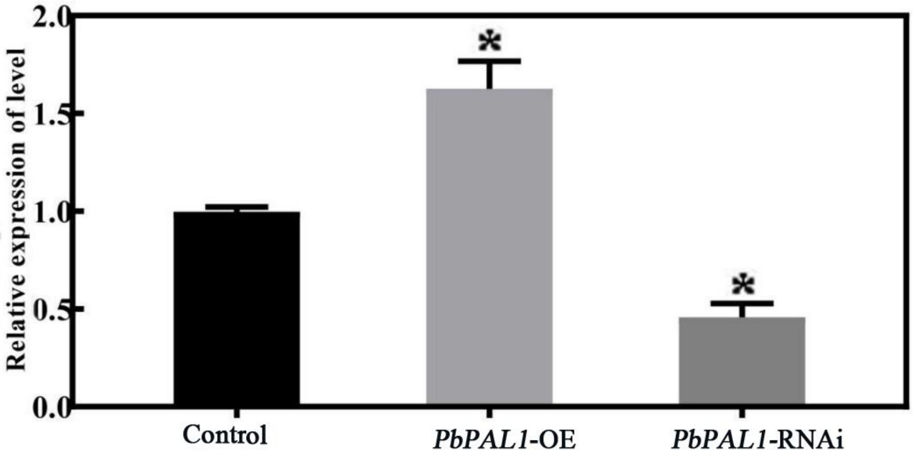

Figure. S4 Analysis of *PbPAL1* expression level in overexpression and RNAi pear fruit
