# Supplementary material for: Functional and kinetics of two efficient phenylalanine ammonia lyase from Pyrus bretschneideri
Source: BMC Plant Biol. 2023 Dec 2;23:612. doi: 10.1186/s12870-023-04586-0 (PMC10693048; doi:10.1186/s12870-023-04586-0)
Supplement: Supplementary file 2 — Additional file 2: Table S1. Potential PbWLIM1 binding sites in the PbPAL promoter. Table S2. Primer sequences contained artificial restriction enzyme sites for Bgl II and Spe I. Table S3. Primer sequences used for qRT-PCR. [file 12870_2023_4586_MOESM2_ESM.pdf]

Additional file:2

Table S1. Potential PbWLIM1 binding sites in the PbPAL promoter

Gene name	Gene ID	Potential LIM binding sites
PbPAL1	Pbr008363.1	/
PbPAL2	Pbr008387.1	CCACCACCATC

Table S2. Primer sequences contained artificial restriction enzyme sites for *Bgl* II and *Spe* I

**Gene name**	**5′→3′**
PbPAL1 Bgl II-F	GA**AGTCT**ATGGAGGCGGAAACCATCAC
PbPAL1 Spe I-R	GC**GTCGAC**CTAACAGATAGGAAGAGGTG
PbPAL2 Bgl II-F	GA**AGTCT**ATGGCTTCTGAGCTAGCTTC
PbPAL2 Spe I-R	GC**GTCGAC**TTAACATATTGGAAGGGGACT

Table S3. Primer sequences used for qRT-PCR

Gene name	Upstream primer （5′→3′）	Downstream primers （5′→3′）
PbPAL1	GAAGTGCTACAGAATCAG	GAATCTTATGCCAGAGTAG
PbPAL2	ATCTCCATCCATCAAGGT	TACAAGGACAGAATGTTCAC
PbPAL3	AAGATTGGAGCTTTCGAGGA	TCTGTTCCAAGCTCTTCCCT
PbC3H	TCAAGCCTGCCTTACAAT	GACGAGGGAACAGTAGTG
Pb4CL1	ACATCAAACAATACATCTCA	ACATCAAACAATACATCTCA
Pb4CL2	CGACAAGGACAACCATAG	GCTCATCATCATCATCAAGA
PbCCoAOMT	CAATTCTTGAACATGCTTCTC	GGAGTAGCCAGTGTAGAC
PbHCT17	ATACCACATTGTGTAGCTGATG	TGCAGAGGGGTTTTTTTAGGG
PbHCT49	GTTAGCGTGAGGGAGTCAACA	GGTCGAAGAACGAGTTGCCA
PbCOMT	TACATCAACTACCACAAGAG	CACAGACCCATTCCATAG
PbCCR2	CGTCCGGAAACAAAGCTAATA	GGTTCCTCTAACAGTGTAGCCTC
PbCAD2	AAGGAAACTGAGGAGATGCTT	TACTTTATTAAATAAGATTGCTG
PbPOD2	GATATTCTCGTCTTGTCTG	TCCTTCCATCTCTTCTTC
PbLAC1	TGGCTTTTCTTCTTGCTCTTATC	CATTGCTGCAGCTGGCAGT
Tublin	AGAACAAGAACTCGTCCTAC	GAACTGCTCGCTCACTCTCC
